# Algorithm for the anesthetic management of cesarean delivery in patients with unsatisfactory labor epidural analgesia

**DOI:** 10.12688/f1000research.6381.1

**Published:** 2015-04-24

**Authors:** Sonia Vaida, Davide Cattano, Debra Hurwitz, Berend Mets

**Affiliations:** 1 Department of Anesthesiology, Penn State Milton S. Hershey Medical Center, Hershey, Pennysylvania, 17033, USA; 2 Preoperative clinic, Department of Anesthesiology, The University of Texas Medical School at Houston, Houston, Texas, 77030, USA

**Keywords:** Epidural labor analgesia, Unsatisfactory labor epidural analgesia, Anesthesia for cesarean delivery

## Abstract

The management of a patient presenting with unsatisfactory labor epidural analgesia poses a severe challenge for the anesthetist wanting to provide safe anesthetic care for a cesarean delivery. Early recognition of unsatisfactory labor analgesia allows for replacement of the epidural catheter. The decision to convert labor epidural analgesia to anesthesia for cesarean delivery is based on the urgency of the cesarean delivery, airway examination, and the existence of a residual sensory and motor block.  We suggest an algorithm which is implemented in our department, based on the urgency of the cesarean delivery.

## Introduction

Neuraxial blockade in obstetric anesthesia is considered the preferred method of analgesia for both vaginal and surgical deliveries. One of the major benefits of labor epidural analgesia is that it can be converted to anesthesia for a surgical delivery if necessary. However, the reported incidence of failure to convert an existing satisfactory labor epidural analgesic to epidural anesthesia for cesarean delivery varies between 1.7%–19.8% ^1, 2^. This large range may be explained by the great variety of techniques used for conversion and the different criteria used for defining failure. In a postal questionnaire of 209 obstetric anesthetists, at least 13 different choices of local anesthetic and adjuvant mixtures used for conversion have been identified ^3^.

Predicting the failure to convert labor epidural analgesia to surgical anesthesia for cesarean delivery is crucial in planning the anesthetic management. Unsatisfactory labor epidural analgesia, which has an incidence of 0.9 to 27% ^1, 4^ may predict a failure to convert to surgical anesthesia ^5, 6^. Unsatisfactory epidural analgesia can be defined as: a unilateral block, unblocked sacral segments, an inadequate block level, a patchy/spotty block, and the persistence of labor pain after the administration of additional local anesthetics or manipulation of the epidural catheter. The reasons given for epidural analgesia failure are: too slow injection of small volumes of local anesthetics, malposition of the epidural catheter, the presence of a congenital median epidural septum, acquired epidural adhesions and adhesion of the dura mater ^7, 10^.

Early recognition of unsatisfactory labor analgesia allows for replacement of the epidural catheter or additional treatment such as the administration of a supplemental mixture of local anesthetics and opioids, with or without partial withdrawing of the epidural catheter ^5, 11– 13^.

Other predictors of failure to convert epidural analgesia to surgical anesthesia include: young age, obesity, higher gestational age at the time of delivery, a higher visual analog scale in the two hours prior to cesarean delivery, the need for more intermittent epidural top-ups during labor, increased maternal height, prolonged labor and anesthetic care being provided by an non-obstetric anesthetist ^2, 10, 13– 17^. An increased failure rate is also associated with a shorter time (under 10 minutes) from decision to perform a cesarean delivery to incision. This is due to insufficient time elapsed from the administration of an epidural top-up to the onset of action of the local anesthetic ^16^. On the other hand, unsatisfactory surgical anesthesia can occur even after satisfactory labor epidural analgesia ^18^.

The anesthetic management of cesarean delivery in a patient found to have an unsatisfactory labor epidural anesthetic depends on the urgency of cesarean delivery. Four categories of urgency are currently defined ^19^:

- Category 1: There is an immediate threat to the life of the mother or the fetus,
- Category 2: There is maternal or fetal compromise which is not immediately life threatening.
- Category 3: There is a need for early delivery but there is no maternal or fetal compromise.
- Category 4: The Cesarean delivery can occur at a time to suit the patient and maternity team.

In this opinion article, we suggest an algorithm to help guide anesthetic management in a situation where epidural analgesia is insufficient and anesthesia is requested for Cesarean delivery.

### Category 1 cesarean delivery ( Figure 1)

The first management decision is based on the airway examination and the anesthetist's assessment as to whether the *in situ* epidural catheter is likely to provide adequate surgical anesthesia. Due to time constraints, this assessment should be based simply on the existence or not of a discernable neuraxial block.

**Figure 1.**
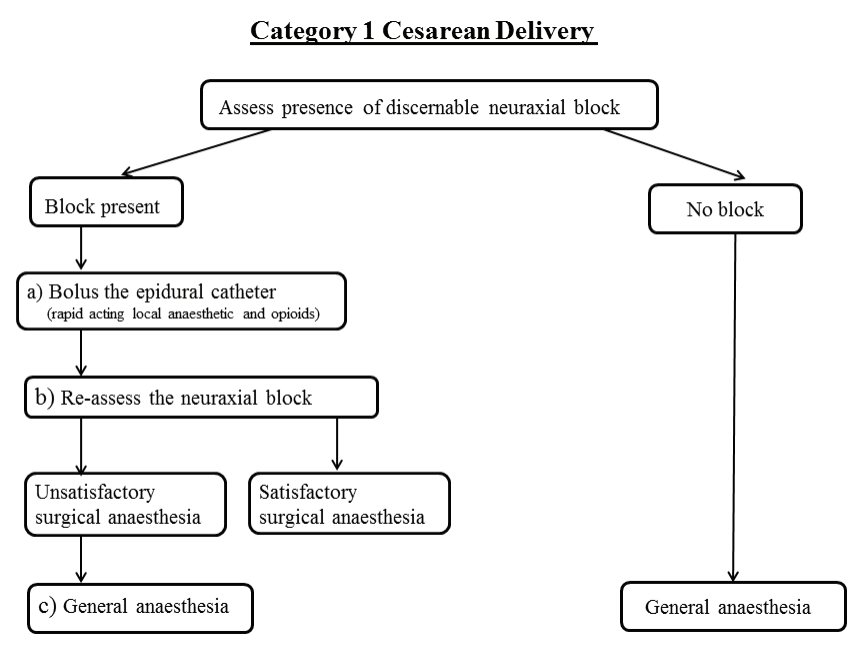
Schematic of Category 1 cesarean delivery algorithm.

If there is a discernable bilateral block, a bolus dose of 15–20 mL of a rapid-acting local anesthetic should be injected though the epidural catheter ( Figure 1a). According to a recent meta-analysis ^20^, a mixture of lidocaine and epinephrine with fentanyl injected into the epidural catheter can achieve one of the fastest sensory blocks in approximately 3 minutes. Alkalinization of the epidural solution with sodium bicarbonate (1mEq/10 mL) can significantly speed the onset of epidural anesthesia ^21^, however it requires increased preparation time ^22^ and can lead to medication errors, especially in the stressful environment of an emergent cesarean delivery ^22^.

Administering a bolus can be justified because administration of supplemental local anesthetic in a higher volume can facilitate spread in the epidural space into previously spared areas ^23^, while carrying a relatively small risk of unintentional intravascular (1:5000) ^23^ or intrathecal injection (1:2900) ^24^. In contrast, administering general anesthesia for cesarean delivery has a much higher risk (1:238) ^25^ of failed intubation. Therefore, especially in patients with predicted difficult airways, every effort should be made to avoid general anesthesia. Preoxygenation and preparation for general anesthesia can begin once the patient is in position, even while dosing the epidural anesthetic.

Given the time constraints, a quick reassessment of the neuraxial block should be performed at the time the surgeon is ready to make the incision ( Figure 1b). Loss of sensation to light touch bilaterally at the dermatomal level of T5 or above is the most reliable method to ensure satisfactory surgical anesthesia. Assessment using other modalities such as loss of sensation to cold and pin-pricks are less reliable ^26, 27^. In addition, before incision, the surgeon should be asked to test the dermatomal level of the neuraxial block by sharp-touch.

If satisfactory epidural surgical anesthesia cannot be achieved, general anesthesia should be induced to guarantee effective surgical anesthesia and cesarean delivery ( Figure 1c). In order to increase the safety of conversion to general anesthesia, the patient’s position should be optimized for intubation, efficient pre-oxygenation performed, and airway backup equipment should be available.

### Category 2 cesarean delivery ( Figure 2)

**Figure 2.**
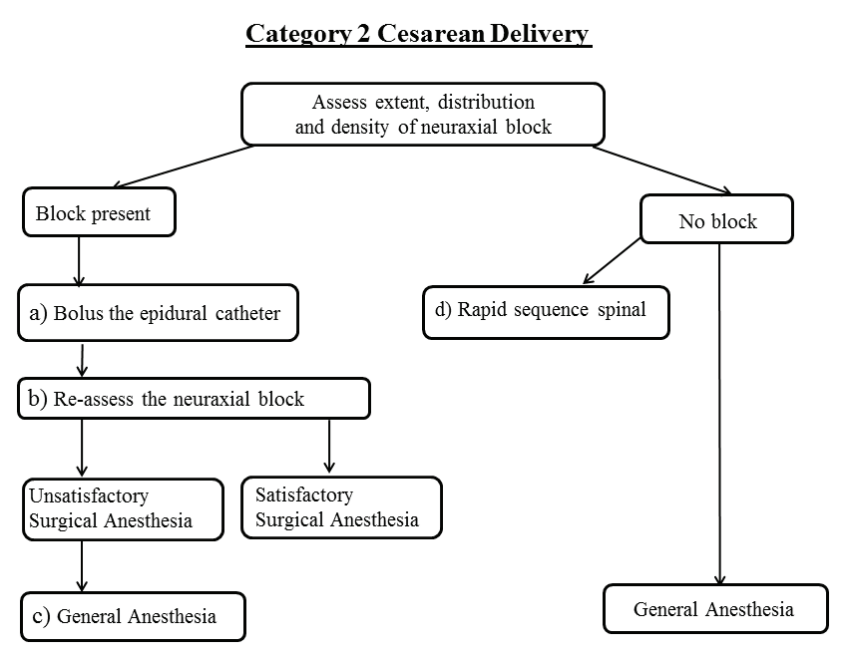
Schematic of Category 2 cesarean delivery algorithm.

A more thorough assessment of the neuraxial block can be performed and should include the height, density and distribution of analgesia (unilateral or bilateral). Should a block be present, fractioned doses of a mixture of local anesthetics and opioids should be administered though the epidural catheter in an attempt to rescue the epidural block ( Figure 2a). Caution should be used not to exceed local anesthetic toxic levels. The epidural block level should be reassessed ( Figure 2b) and if found to be unsatisfactory, an immediate conversion to general anesthesia should occur ( Figure 2c).

In patients with no evidence of neuraxial blockade, spinal anesthesia may be considered ( Figure 2d), as this has been described to be almost as quick as a general anesthetic in experienced hands. Kinsella *et al.* ^28^ described a “rapid sequence spinal” technique; a non-touch technique allowing only limited attempts, no local skin infiltration, and necessitating 8 minutes (on average) to complete. This is a newly described and not yet universally accepted technique. Criticisms of this “rapid sequence spinal” technique include the lack of aseptic preparation and the inability to establish a prior rapport in an anxious patient ^29, 30^.

Alternatively general anesthesia could be used, as this is the quickest approach to reliably anesthetize the patient for cesarean delivery. Clinical situations that would favor immediate conversion to general anesthesia include the presence of an analgesic window; neuraxial dermatomal levels below T12; a unilateral block that differs by more than two or three dermatomal levels or insufficient analgesic density with uneven distribution of numbness to soft touch.

### Category 3 and 4 cesarean delivery ( Figure 3)

In a situation where there is no maternal or fetal compromise, a complete evaluation of the degree of the motor and sensory block can be performed. Epidural sensory block should be assessed for the highest dermatomal level at which the patient is able to detect a change in sensation to light touch, bilateral distribution and potential analgesic windows. The degree of epidural motor block can be assessed using a modified Bromage score (1 = able to raise legs above table, 2 = able to flex knees, 3 = able to move feet only, 4 = no movement in legs or feet).

**Figure 3.**
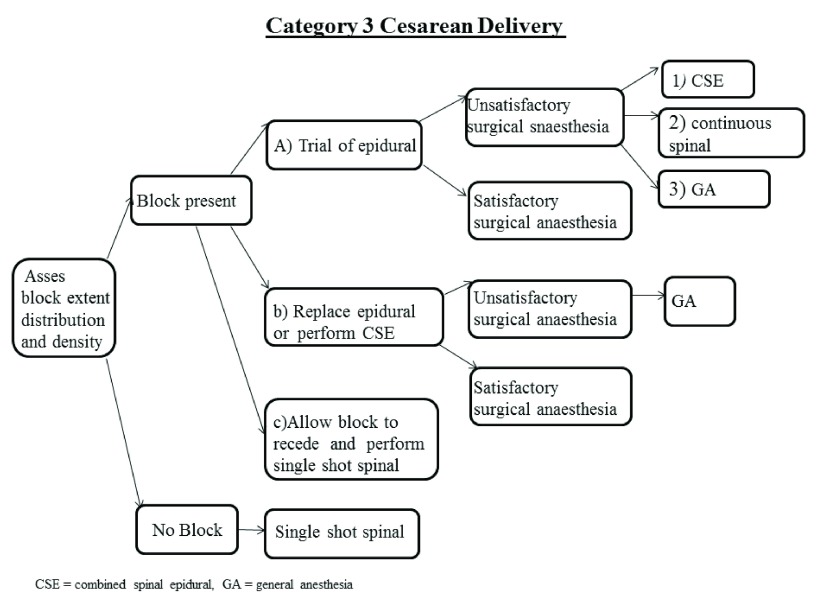
Schematic of Category 3 and 4 cesarean delivery algorithm.

In patients with residual block there are three management options:

***Option A – Trial of epidural* ( Figure 3a).** The most common situation encountered in clinical practice is a patient with some degree of sensory and/or motor block. In this situation, fractioned epidural administration of one quarter to one third of the final anticipated dose is indicated in order to ascertain whether the anesthesia will be effective before injecting the entire dose. If this trial of epidural fails, one can proceed to either a combined spinal epidural (CSE) anesthetic ( Figure 3-1), continuous spinal ( Figure 3-2), or general anesthesia ( Figure 3-3).

A CSE allows the use of a lower intrathecal dose with the additional flexibility of supplementation of the block through an epidural catheter. Portnoy and Valdhera ^10^ recommend decreasing the dose of local anesthetic injected intrathecally by 20–30% to avoid a high spinal block. Intrathecal doses of bupivacaine as low as 4.5–6.5 mg have been used successfully for cesarean delivery ^31, 32^. A standard dose spinal anesthetic at this stage could result in an unpredictable cephalad extension of the neuraxial block ( *vide infra*) ^33^.

Although controversial, a continuous spinal catheter is a viable option for cesarean delivery after failed epidural analgesia ^34^. The advantage of a continuous spinal technique lies in the immediate confirmation of a successful block, and the ability to use careful titration of local anesthetics in boluses or as a continuous infusion. 5.0 mg of 0.5% preservative-free bupivacaine, plus 15 µg fentanyl can be initially injected intrathecally, followed by 2.5 mg boluses of 0.5% bupivacaine every 5 minutes until a T5 dermatomal level is achieved ^34^.

General anesthesia would assure a reliable anesthetic for cesarean delivery.

***Option B - Remove the epidural catheter and perform a CSE or a* de novo *epidural*** **( Figure 3b).** An alternative to option A is to remove the epidural catheter as soon as the decision to perform a cesarean delivery is made and then replace the epidural catheter or administer a CSE. This option should be considered especially in patients where previous attempts to rescue labor epidural analgesia have been performed (withdrawal of the epidural catheter by 1 cm followed by top-up with local anesthetics and opioids).

***Option C - Allow the residual block to recede and perform a single shot spinal anesthetic*** **( Figure 3c).** This option may be recommended especially in patients presenting with unilateral block and a very high level of sensory block on one side. One should wait until the residual epidural block wears off before a spinal anesthetic is performed. It has been recommended to wait at least 30 minutes after the last epidural bolus before initiating a spinal anesthetic ^10^. This is because there is an associated risk of a subsequent high block especially after administration of a recent epidural bolus just prior to a spinal anesthetic ^20, 35^. The described cephalad spread of the block can result from the compression of the spinal space by the previously injected epidural solution and/or the leakage of the epidural solution into the intrathecal space ^10^.

General anesthesia may become the preferred choice if the mother or fetus’ condition change, the total dose of local anesthetic administered approaches the potential for toxicity, or after multiple failed attempts at neuraxial anesthesia.

In patients with no residual block a spinal anesthetic can be safely performed.

## Summary

In summary, the management of a patient with unsatisfactory labor epidural analgesia poses a severe challenge for the anesthetist wanting to provide safe anesthetic care for a cesarean delivery. Good communication with the obstetric team to anticipate a cesarean delivery will allow for adequate planning for safe conversion of unsatisfactory epidural analgesia to adequate surgical anesthesia.
