# Supplementary material for: Algorithm for the anesthetic management of cesarean delivery in patients with unsatisfactory labor epidural analgesia
Source: F1000Res. 2015 Apr 24;4:98. [Version 1] doi: 10.12688/f1000research.6381.1 (PMC4482209; doi:10.12688/f1000research.6381.1)
Supplement: Peer review history

# Peer review: Referee response for version 1

**DOI:** 10.5256/f1000research.6843.r8960

**Published:** 2015-06-18

**Authors:** Matteo Parotto

**Affiliations:** 1 Department of Anesthesia, University of Toronto, Toronto, ON, Canada

This very interesting opinion article provides a proposal from the Authors of an algorithm to help guide the anesthetic management of patients with unsatisfactory labor epidural analgesia that require cesarean delivery. It is a clear, concise, substantiated suggestion on how to approach a difficult clinical scenario.

I feel it would be useful for the reader to receive more specific information/author's opinion on the definition of analgesic window and its clinical implications, and on the doses of anesthetics chosen in the various situations described.

There might be a typo in the title for Figure 3, which may be changed to "Category 3 and 4 Cesarean Delivery".

I have read this submission. I believe that I have an appropriate level of expertise to confirm that it is of an acceptable scientific standard.

# Peer review: Referee response for version 1

**DOI:** 10.5256/f1000research.6843.r8455

**Published:** 2015-05-05

**Authors:** Wanda Popescu

**Affiliations:** 1 Department of Anesthesiology, Yale University, New Haven, CT, USA

The article provides a very good algorithm to follow in case a patient with an unsatisfactory epidural requires anesthesia for a C-section. For completeness I would suggest that the authors be more specific in describing the medications used in different situations of blocks (exact medication, recommended concentration and dose). I would also recommend that the authors be slightly more detailed when describing the "rapid sequence spinal", in particular as this appears to be a rather new and controversial topic. Otherwise it's a great article.

I have read this submission. I believe that I have an appropriate level of expertise to confirm that it is of an acceptable scientific standard.
